# Association of ambient air pollution with hemoglobin levels and anemia in the general population of Korean adults

**DOI:** 10.1186/s12889-024-18492-z

**Published:** 2024-04-09

**Authors:** Juyeon Hwang, Hyun-Jin Kim

**Affiliations:** Cancer Big Data Center, National Cancer Control Institute, National Cancer Center, 323 Ilsan-ro, Ilsandong-gu, 10408 Goyang-si Gyeonggi-do, South Korea

**Keywords:** Ambient air pollution, Chronic exposure, Hemoglobin level, Anemia, General population, Adults

## Abstract

**Background:**

Emerging evidence has suggested significant associations between ambient air pollution and changes in hemoglobin levels or anemia in specific vulnerable groups, but few studies have assessed this relationship in the general population. This study aimed to evaluate the association between long-term exposure to air pollution and hemoglobin concentrations or anemia in general adults in South Korea.

**Methods:**

A total of 69,830 Korean adults from a large-scale nationwide survey were selected for our final analysis. Air pollutants included particulate matter with an aerodynamic diameter less than or equal to 10 micrometers (PM_10_), particulate matter with an aerodynamic diameter less than or equal to 2.5 micrometers, nitrogen dioxide, sulfur dioxide (SO_2_), and carbon monoxide (CO). We measured the serum hemoglobin concentration to assess anemia for each participant.

**Results:**

In the fully adjusted model, exposure levels to PM_10_, SO_2_, and CO for one and two years were significantly associated with decreased hemoglobin concentrations (all *p* < 0.05), with effects ranging from 0.15 to 0.62% per increase in interquartile range (IQR) for each air pollutant. We also showed a significant association of annual exposure to PM_10_ with anemia (*p* = 0.0426); the odds ratio (OR) [95% confidence interval (CI)] for anemia per each increase in IQR in PM_10_ was estimated to be 1.039 (1.001–1.079). This association was also found in the 2-year duration of exposure (OR = 1.046; 95% CI = 1.009–1.083; adjusted Model 2). In addition, CO exposure during two years was closely related to anemia (OR = 1.046; 95% CI = 1.004–1.091; adjusted Model 2).

**Conclusions:**

This study provides the first evidence that long-term exposure to air pollution, especially PM_10_, is significantly associated with reduced hemoglobin levels and anemia in the general adult population.

**Supplementary Information:**

The online version contains supplementary material available at 10.1186/s12889-024-18492-z.

## Background

Anemia, a blood disorder of insufficient hemoglobin or red blood cells (RBCs), is a major health issue around the world, contributing to negative health outcomes, increased risk of morbidity, and substantial economic costs [1]. According to a recent report in 2021, the global prevalence of anemia at all ages is estimated to be 24.3%, corresponding to 1.92 billion patients [2]. In particular, it has been well-reported that anemia is more prevalent in children, pregnant women, and the elderly, and that it leads to a higher risk of poor clinical outcomes, including impaired cognitive function, cardiovascular disease, and mortality [3–5]. Therefore, previous epidemiological studies to identify risk factors associated with anemia have focused mainly on these vulnerable groups and have found several risk factors such as low socioeconomic level, use of smoke-producing fuels, comorbidities, childhood malnutrition, and maternal anemia [6–8].

In recent years, a growing body of evidence has also shown the detrimental effects of air pollution exposure on hemoglobin concentrations and anemia in specific populations (i.e., children, pregnant women, and the elderly) [9–13]. Ambient air pollution, which is a complex mixture of liquid droplets, gases, and solid particles, contributes to disturbances in iron homeostasis [14]. Exposure to air pollution leads to cellular iron deficiency through the activation of oxidant production and increased secretion of pro-inflammatory mediators. Furthermore, increased pro-inflammatory cytokines caused by exposure to air pollution can lead to a deficiency in erythropoietin secretion, resulting in anemia [15–18]. In addition, exposure to air pollution increases the secretion of reactive oxygen species, resulting in oxidative stress. In 2008, an experimental study in a murine model reported that oxidative stress was closely related to iron deficiency anemia [19]. These molecular pathways, such as inflammation and oxidative stress, can be relevant in the adults and vulnerable groups mentioned above.

Although previous reports have identified significant associations between air pollution and anemia in limited and vulnerable samples or specific pollutants such as particulate matter (PM), more evidence is needed to better understand these relationships in the general population. However, to our knowledge, research has yet to be performed to evaluate these associations in the general adult population. Therefore, this study aimed to investigate the relationship between long-term exposure to ambient air pollution and hemoglobin levels or anemia in representative general adults in South Korea.

## Methods

### Study population

**Fig. 1:**
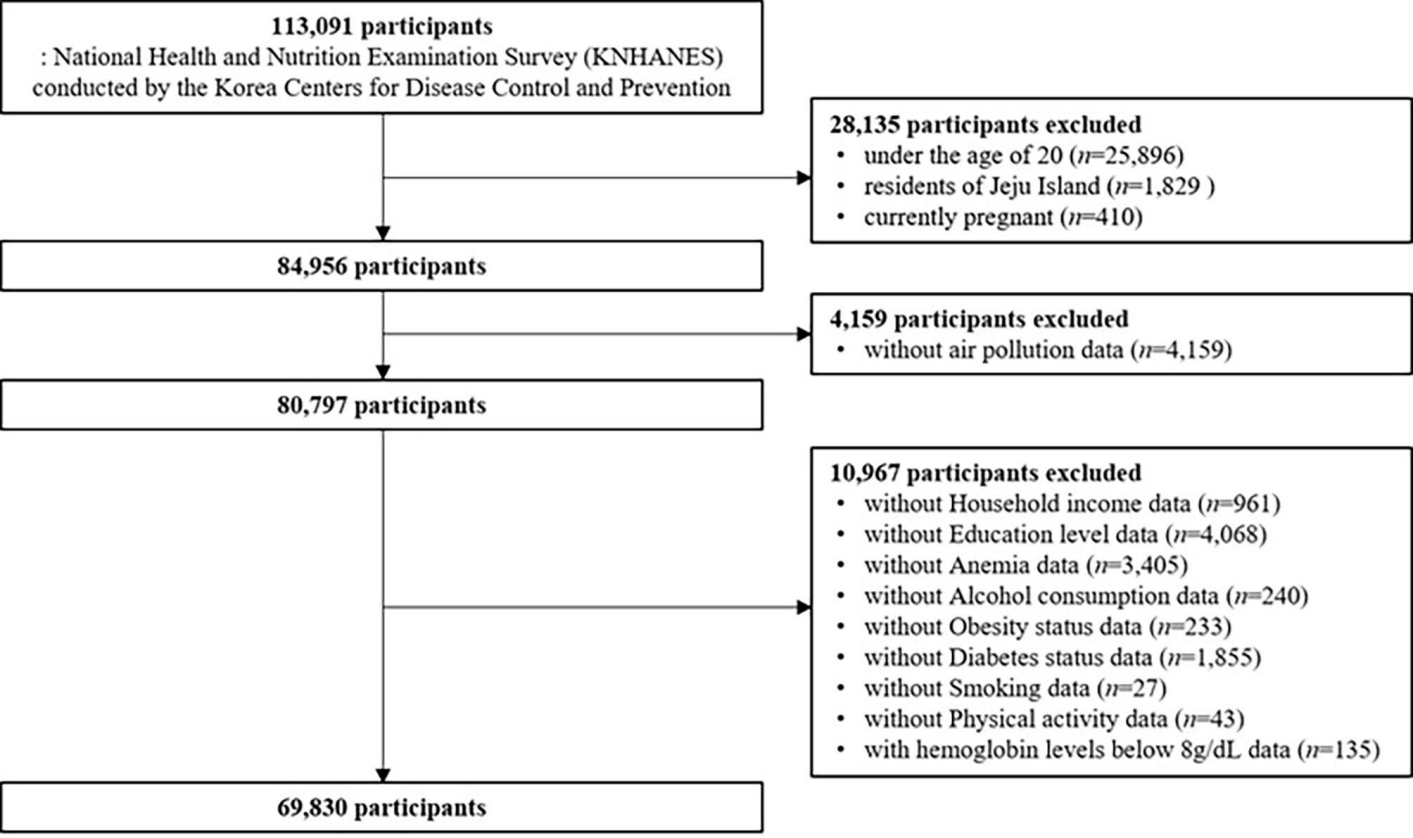
Diagram illustrating participant selection

The participants in our study were recruited from the National Health and Nutrition Examination Survey (KNHANES) conducted by the Korean Centers for Disease Control and Prevention to collect nationally representative data on risk factors and diseases and identify target groups at risk. KNHANES is an ongoing national surveillance system that was launched in 1998 and has been conducting investigations ever since. Briefly, this survey collects various data on health and nutritional status, such as socioeconomic status, health-related behaviors, and clinical profiles. This study included 14 years of survey data (2007–2020) when ambient air pollution data were available. A total of 113,091 subjects participated in the abovementioned survey period, and only 69,830 of whom met the following exclusion criteria were finally considered in our study: (1) general adults aged < 20 years (for capturing a representative sample of the general adult population), residents of Jeju Island, or currently pregnant (*n* = 28,135); (2) those without estimated exposure concentrations to ambient air pollution (*n* = 4,159); (3) those whose records included hemoglobin levels (*n* = 135); and (4) those who provided accurate information about variables of interest such as socioeconomic status and health behaviors (*n* = 10,832). A detailed diagram illustrating participant selection is shown in Fig. 1. The KNHANES was approved by the Institutional Review Board of the Korea Centers for Disease Control (IRB No. 1401–047–547), and all participants signed an informed consent form. This study meets the ethical principles based on the Declaration of Helsinki for medical research involving human subjects.

### Measurement of air pollution exposure

The Community Multiscale Air Quality (CMAQ, v4.7.1) model developed by the US Environmental Protection Agency was considered based on modeling data for weather and emissions to estimate exposure to air pollution. Meteorological data were obtained from the National Centers for Environmental Prediction and the Global Forecast System, and Weather Research and Forecasting v3.6.1 was used for modeling of these data. The modeling of emissions data was also performed with Sparse Matrix Operator Kernel Emissions v2.7 using relevant data, including domestic Clean Air Policy Support System, Multiresolution Emission Inventory for China, and Regional Emission Inventory in Asia. For the efficiency of modeling, initial estimates were calculated using the NESTING technique for East Asia and the domestic region, applying grid units of 27 km and 9 km, respectively. To improve accuracy, the initial concentration values estimated by CMAQ were recalculated by data assimilation. Data assimilation is a method of recalculating the accuracy of the initial value calculated through CMAQ modeling using the values of all observations within the grid. For data assimilation, air quality measurement data from China and Korea were used, and the number of measurement stations was 1,496 and 323, respectively. Air pollutants include particulate matter ≤ 2.5 μm in diameter (PM_2.5_), particulate matter ≤ 10 μm in diameter (PM_10_), nitrogen dioxide (NO_2_), sulfur dioxide (SO_2_), and carbon monoxide (CO). In particular, in the case of PM, a multiple regression analysis was performed using various data related to PM_2.5_ and PM_10_ (e.g., temperature, humidity, wind speed, barometric pressure, and air quality) as well as satellite-based aerosol optical depth (AOD), and PM such as PM_2.5_ and PM_10_ was finally calculated on a 1-km grid unit. For AOD data, MCD19A2 AOD (1 km, daily unit) and MODIS L3 vegetation index (NDVI), which applied the Multi-angle Implementation of Atmospheric Correction (MAIAC) algorithm with data measured from the Moderate Resolution Imaging Spectroradiometer (MODIS) sensor, were used. For validation, the concentrations estimated through modeling were compared with the concentrations at the air quality measurement data. This study included the KNHANES data from 2007 to 2020. On the other hand, modeling data on air pollution concentrations were estimated from 2005 to 2017. Therefore, the exposure period was finally considered as 1-year and 2-years to use all air pollution modeling data.

### Evaluation of hemoglobin levels or anemia status and variables of interest

We evaluated hemoglobin levels from standard blood samples taken after a minimum of 8 h of fasting. Serum hemoglobin levels (g/dL) were measured using an XE-2100D (Sysmex Corp., Kobe, Japan). We classified subjects into two groups according to the criteria of the World Health Organization for anemia as follows: anemia (hemoglobin < 13 g/dL for men and < 12 g/dL for women) and normal (hemoglobin ≥ 13 g/dL for men and ≥ 12 g/dL for women). We included potential confounding variables such as demographic information, health-related behaviors, anthropometric measurements, and medical conditions to investigate the associations between exposure to air pollutants and hemoglobin levels or anemia status. Demographic information (e.g., age, sex, education level, and income) and lifestyle behaviors (e.g., smoking, alcohol consumption, and physical activity) were investigated by a structured questionnaire. Physical activity was defined as moderate activities on at least three or more days a week [20]. Smoking status was classified into two group (never- or former-smoker vs. current smoker) and alcohol consumption was divided into four groups according to the frequency per month (never, less than or equal to one time, 2 to 15 times, or more than 16 times). We also obtained the anthropometric measurements, such as height and weight, and calculated the body mass index (BMI) by dividing weight (kg) by the square of height (m^2^). Participants were classified into three groups according to their BMI level as follows: normal weight (BMI < 25 kg/m^2^), overweight (25 kg/m^2^ ≤ BMI < 30 kg/m^2^), and obesity (BMI ≥ 30 kg/m^2^) [21]. Hypertension was defined as systolic blood pressure ≥ 140 mmHg, diastolic blood pressure ≥ 90 mmHg, or taking antihypertensive medications [22]. Furthermore, diabetes was defined as a fasting blood sugar level of 126 mg/dL or higher or taking diabetic medications or receiving insulin therapy [23]. However, unfortunately, we could not include information regarding iron supplements or dietary intake, due to the absence of relevant data.

### Statistical analysis

Before analysis, we checked the distribution of hemoglobin concentrations to identify the normality assumption. Because hemoglobin levels did not follow a normal distribution, the values were finally logarithmically transformed to approximate a normal distribution. The t-test and the chi-square test were performed to evaluate differences in characteristics between the anemia and normal groups. Multiple linear regression analysis was performed to identify the association between ambient air pollution and hemoglobin levels; the association results are shown as beta coefficients (*β*s) and 95% confidence intervals (CIs) for hemoglobin levels. Furthermore, we performed multiple logistic regression analysis to assess the association between exposure to air pollutants and the presence of anemia; the association results are shown as odds ratios (ORs) and 95% CIs for each air pollutant. These estimates (i.e., *β*s and ORs) were converted to an interquartile range (IQR) scale for each air pollutant [8.3 (7.4) micrograms per cubic meter (µg/m^3^) for 1-year (2-year) exposure to PM_10_, 4.6 (4.1) µg/m^3^ for 1-year (2-year) exposure to PM_2.5_, 16.1 (16.2) parts per billion (ppb) for 1-year (2-year) exposure to NO_2_, 1.7 (1.6) ppb for 1-year (2-year) exposure to SO_2_, and 123.6 (123.7) ppb for 1-year (2-year) exposure to CO]. We first considered various variables such as demographic information, health-related behaviors, anthropometric measurements, and medical conditions as potential covariates, and finally selected variables with significant different between anemia and normal groups as covariates in the statistical model. As shown in Table 1, all potential variables showed significant statistical differences between the two groups, and therefore all of them were selected as covariates. These associations were presented in crude and two adjusted models (adjusted Models 1 and 2). Model 1 included confounding factors such as age, sex, household income, education level, place of residence (urban vs. rural), alcohol consumption, smoking status, physical activity, and occupation. Model 2 adjusted Model 1 plus medical conditions, including hypertension status, diabetes status, and obesity status (normal, overweight, and obesity). We also performed a stratified association analysis by sex. In addition, the propensity score matching (PSM) analysis was utilized to address group imbalances. All statistical analyses were performed using statistical analysis software (SAS) version 9.4 (SAS Institute, Cary, NC, USA).

**Table 1:** Characteristics of the study population depending on anemia

Characteristics	Anemia (Women: Hemoglobin level < 12 g/dL) (Men: Hemoglobin level < 13 g/dL)	Normal (Women: Hemoglobin level ≥ 12 g/dL) (Men: Hemoglobin level ≥ 13 g/dL)	*p*
	Mean ± SD or n (%)	Mean ± SD or n (%)	
***n***	6,248 (9.0)	63,582 (91.1)	
**Age**(year)	54.2 ± 17.2	50.1 ± 16.0	< 0.0001
**Sex**			< 0.0001
Women	4,937 (79.0)	34,566 (54.4)	
Men	1,311 (21.0)	29,016 (45.6)	
**Education level**			< 0.0001
Less than elementary school	1,929 (30.9)	13,951 (21.9)	
Middle school	624 (10.0)	6,777 (10.7)	
High school	1,911 (30.6)	21,347 (33.6)	
College or graduate school	1,784 (28.6)	21,507 (33.8)	
**Residential region**			< 0.0001
Rural	1,952 (31.2)	17,628 (27.7)	
Urban	4,296 (68.8)	45,954 (72.3)	
**Smoking**			< 0.0001
Never or Former-smokers	5,558 (89.0)	47,553 (74.8)	
Current smokers	690 (11.0)	16,029 (25.2)	
**Alcohol consumption** (time/month)			< 0.0001
Never	2,497 (40.0)	16,869 (26.5)	
≤ 1	1,949 (31.2)	18,102 (28.5)	
2 ∼ 15	1,572 (25.2)	24,056 (37.8)	
≥ 16	230 (3.7)	4,555 (7.2)	
**Physical activity**			< 0.0001
Yes	1,325 (21.2)	15,704 (24.7)	
No	4,923 (78.8)	47,878 (75.3)	
**Household income** (Quartile, Thousand KRW)			< 0.0001
Low (< 76)	1,623 (26.0)	11,392 (17.9)	
Lower-medium (76 ∼ 154)	1,583 (25.3)	15,837 (24.9)	
Upper-medium (155 ∼ 253)	1,598 (25.6)	17,536 (27.6)	
High (≥ 254)	1,444 (23.1)	18,817 (29.6)	
**Occupational group**			< 0.0001
Office worker	614 (9.8)	8,207 (12.9)	
Service/sales worker	416 (6.7)	4,644 (7.3)	
Skilled agricultural, forestry, fishery workers	282 (4.5)	2,684 (4.2)	
Craft and related trades workers/plant, machine operators and assemblers/elementary workers	514 (8.2)	7,030 (11.1)	
Unemployed/unknown	4,422 (70.8)	41,017 (64.5)	
**Body mass index** (kg/m^2^)	22.9 ± 3.3	23.9 ± 3.4	< 0.0001
**Obesity status**			< 0.0001
Normal	4,771 (76.4)	41,828 (65.8)	
Overweight	1,292 (20.7)	18,739 (29.5)	
Obesity	185 (3.0)	3,015 (4.7)	
**Hypertension status**			< 0.0001
Yes	2,114 (33.8)	19,696 (31.0)	
No	4,134 (66.2)	43,886 (69.0)	
**Diabetes status**			< 0.0001
Yes	1,008 (16.1)	7,106 (11.2)	
No	5,240 (83.9)	56,476 (88.8)	
**Hemoglobin level** (g/dL)	11.3 ± 0.9	14.3 ± 1.3	< 0.0001

SD, standard deviation

## Results

### Characteristics of the participants according to anemic and normal groups

The study characteristics of the subjects stratified by anemic (*n* = 6,248) and normal (*n* = 63,582) groups are presented in Table 1. The anemic group was slightly older (54.2 years) than the normal group (50.1 years), and the anemic participants had a higher percentage of women. Regarding the level of education, the anemic group had the highest proportion of individuals with elementary school education or less (30.9%). In comparison, the highest proportion in the normal group had college or higher education (33.8%). Both groups live primarily in urban areas. The proportion of current smokers was significantly higher in the normal group (25.2%) than in the anemic group (11.0%). The normal group had a higher monthly alcohol consumption than the anemic participants. The proportion of subjects who were overweight or obese was much higher in the normal group (34.2%) than in the anemic group (23.6%). By contrast, there were more patients with hypertension or diabetes in the anemic group than in the normal group. The mean hemoglobin levels in the anemic and normal groups were 11.3 and 14.3 g/dL, respectively. Besides, the hemoglobin levels increased as the years increased, but decreased largely in 2020 (Figure S1). Similarly, the prevalence of anemia showed a gradual decreasing trend since 2007, but then increased again in 2020. In addition, the distribution maps of air pollution concentration and anemia incidence rate by region for the most recent year (2020) were demonstrated in Figure S2.

### Distribution of air pollutants by exposure periods (1- and 2-year exposures)

Table 2 shows the distributions of five air pollutants for 1- and 2-year exposure durations. The mean values for PM_10_, PM_2.5_, SO_2_, NO_2_, and CO concentrations during 1-year exposure were 49.4 µg/m^3^, 24.2 µg/m^3^, 4.7 ppb, 25.3 ppb, and 486.5 ppb, respectively. The median values (IQR) for PM_10_, PM_2.5_, SO_2_, NO_2_, and CO concentrations during 1 year of exposure were 48.9 (8.3) µg/m^3^, 23.9 (4.6) µg/m^3^, 4.4 (1.7) ppb, 23.4 (16.1) ppb, and 489.1 (123.6) ppb, respectively. Each pollutant was statistically significantly correlated with each other (0.1 < *r* < 0.8, all *p* < 0.0001). The distribution of each air pollutant during 2 years of exposure and their correlation results were similar to those of 1 year of exposure. As shown in Figure S1, as the year increased, the average concentration values of PM (i.e. PM_2.5_ and PM_10_) and SO_2_ exposures gradually decreased.

**Table 2:** The distributions of air pollutants by exposure duration (1-year and 2-years)

Air pollutants	Mean ± SD	Median	IQR	Pearson’s correlation coefficients				
				PM_10_	PM_2.5_	SO_2_	NO_2_	CO
**1-year**								
PM_10_ (µg/m^3^)	49.4 ± 6.6	48.9	8.3	1	0.8*	0.4*	0.5*	0.6*
PM_2.5_ (µg/m^3^)	24.2 ± 3.7	23.9	4.6	0.8*	1	0.3*	0.1*	0.4*
SO_2_ (ppb)	4.7 ± 1.9	4.4	1.7	0.4*	0.3*	1	0.3*	0.3*
NO_2_ (ppb)	25.3 ± 9.9	23.4	16.1	0.5*	0.1*	0.3*	1	0.6*
CO (ppb)	486.5 ± 84.9	489.1	123.6	0.6*	0.4*	0.3*	0.6*	1
**2-years**								
PM_10_ (µg/m^3^)	50.0 ± 6.3	49.4	7.4	1	0.8*	0.4*	0.5*	0.7*
PM_2.5_ (µg/m^3^)	24.5 ± 3.5	24.2	4.1	0.8*	1	0.3	0.1*	0.4*
SO_2_ (ppb)	4.8 ± 1.9	4.5	1.6	0.4*	0.3*	1	0.3*	0.3*
NO_2_ (ppb)	25.2 ± 9.8	23.4	16.2	0.5*	0.1*	0.3*	1	0.6*
CO (ppb)	491.9 ± 85.1	496.3	123.7	0.7*	0.4*	0.3*	0.6*	1

SD, standard deviation; IQR, interquartile range; PM_10,_ particulate matter ≤ 10 μm in diameter; PM_2.5,_ particulate matter ≤ 2.5 μm in diameter; SO_2_, sulfur dioxide; NO_2_, nitrogen dioxide; CO, carbon monoxide

*p < 0.0001

### Association between ambient air pollution and hemoglobin level

Linear regression analyses in crude and adjusted models were performed to evaluate the association between exposure to air pollution and hemoglobin concentration (Table 3). In the case of annual mean levels of air pollutants, exposures to PM_2.5_ and NO_2_ in the crude model showed significant positive associations with hemoglobin levels, but these associations disappeared after adjusting for confounding variables. In both adjusted models, exposure to PM_10_ was significantly associated with a lower hemoglobin concentration (*p* < 0.05); there was a 0.43% decrease in hemoglobin level with an IQR (8.3 µg/m^3^) increase in PM_10_ (95% CI = − 0.59%, − 0.27%) in adjusted Model 2. In adjusted models, exposure to SO_2_ and CO was also associated with decreased hemoglobin levels; hemoglobin values decreased by 0.15% (β = − 0.0015; 95% CI = − 0.0020, − 0.0009) and 0.54% (β = − 0.0054; 95% CI = − 0.0072, − 0.0036) for each increase in IQR in SO_2_ and CO concentration, respectively (adjusted Model 2). The pattern of association results during 2 years of exposure was similar to that of 1 year of exposure. After stratification by sex, there was no significant difference in association results between women and men (Table S1). In both women and men, exposures to PM_10_, SO_2_, and CO were significantly associated with decreased hemoglobin levels. Notably, a significant association between NO_2_ exposure and decreased hemoglobin levels was found only in men (β = − 0.0062 for 1 year of exposure; 95% CI = − 0.0097, − 0.0027).

**Table 3:** Association between hemoglobin levels (g/dL) and interquartile range (IQR) in annual average air pollution exposure

Exposure	Log (Hemoglobin level)					
	Crude model		Adjusted model 1^†^		Adjusted model 2^† †^	
	β (95% CI)	*p*	β (95% CI)	*p*	β (95% CI)	*p*
**1-year**						
PM_10_ (µg/m^3^)	-0.0007 (-0.0028, 0.0015)	0.5313	-0.0047 (-0.0063, -0.003)	< 0.0001	-0.0043 (-0.0059, -0.0027)	< 0.0001
PM_2.5_ (µg/m^3^)	0.0024 (0.0007, 0.004)	0.0059	0.0003 (-0.0009, 0.00158)	0.6128	0.0006 (-0.0007, 0.0018)	0.3566
SO_2_ (ppb)	-0.0011 (-0.002, -0.0003)	0.0053	-0.0016 (-0.0022, -0.001)	< 0.0001	-0.0015 (-0.002, -0.0009)	< 0.0001
NO_2_ (ppb)	0.0031 (0.0007, 0.0057)	0.0145	0.0000 (-0.0025, 0.0024)	0.9678	0.0000 (-0.0023, 0.0025)	0.9432
CO (ppb)	-0.0001 (-0.0024, 0.0021)	0.9071	-0.0058 (-0.0076. -0.0039)	< 0.0001	-0.0054 (-0.0072, -0.0036)	< 0.0001
**2-years**						
PM_10_ (µg/m^3^)	-0.0016 (-0.0036, 0.0004)	0.1121	-0.0056 (-0.0072, -0.0041)	< 0.0001	-0.0054 (-0.0069, -0.0038)	< 0.0001
PM_2.5_ (µg/m^3^)	0.0024 (0.0006, 0.0042)	0.008	0.0000 (-0.0014, 0.0013)	0.9833	0.0002 (-0.0011, 0.0016)	0.7361
SO_2_ (ppb)	-0.0012 (-0.002, -0.0004)	0.0051	-0.0017 (-0.0023, -0.0011)	< 0.0001	-0.0016 (-0.0023, -0.001)	< 0.0001
NO_2_ (ppb)	0.0029 (0.0004, 0.0054)	0.0264	-0.0004 (-0.0028, 0.002)	0.7509	-0.0003 (-0.0027, 0.0021)	0.8019
CO (ppb)	-0.0006 (-0.0029, 0.0016)	0.5895	-0.0065 (-0.0083, -0.0047)	< 0.0001	-0.0062 (-0.008, -0.0044)	< 0.0001

PM_10,_ particulate matter ≤ 10 μm in diameter; PM_2.5,_ particulate matter ≤ 2.5 μm in diameter; SO_2_, sulfur dioxide; NO_2_, nitrogen dioxide; CO, carbon monoxide; CI: confidence interval

†Adjusted model 1 includes the following variables: place of residence (urban vs. rural), age, sex, household income, education level, alcohol consumption, smoking status, physical activity, and occupation

††Adjusted model 2 includes the following variables: place of residence (urban vs. rural), age, sex, household income, education level, alcohol consumption, smoking status, physical activity, occupation, hypertension status, diabetes status, and obesity status (normal, overweight and obesity)

### Association between ambient air pollution and anemia

We performed simple and multiple logistic regression analyses to examine the association between air pollution exposure and anemia, and the results are shown in Table 4. In the crude model, most of the air pollutants, including PM_10_, PM_2.5_, NO_2_, and CO at 1 and 2 years of exposure, were inversely related to anemia. However, after adjusting for covariates, the statistical significance of the association either disappeared or changed to a positive association, except for exposure to NO_2_. In adjusted Model 2, only exposure to PM_10_ was significantly associated with an increased risk of anemia (*p* = 0.0426); OR (95% CIs) for anemia per each increase in IQR in PM_10_ was estimated at 1.039 (1.001–1.079). A positive association was also found between exposure to PM_10_ and anemia in the 2-year duration of exposure (OR = 1.046; 95% CI = 1.009–1.083; adjusted Model 2). The results of the 2-year exposure period showed a significant positive association between CO exposure and anemia in adjusted models (OR = 1.046; 95% CI = 1.004–1.091; adjusted Model 2). However, PM_2.5_ and SO_2_ exposures did not show a significant association with anemia, similar to the results of the 1-year exposure period. In the stratification results by sex, a positive association was only found between exposure to PM_10_ and anemia in the 2-year duration of exposure in women (OR = 1.044; 95% CI = 1.003–1.086; adjusted Model 2) (Table S2).

**Table 4:** Association between anemia status and interquartile range (IQR) in annual average air pollution exposure

Exposure	Anemia status					
	Crude model		Adjusted model 1^†^		Adjusted model 2^† †^	
	OR (95% CI)	*p*	OR (95% CI)	*p*	OR (95% CI)	*p*
**1-year**						
PM_10_ (µg/m^3^)	0.964 (0.93, 0.999)	0.0448	1.039 (1.001, 1.078)	0.0428	1.039 (1.001, 1.079)	0.0426
PM_2.5_ (µg/m^3^)	0.969 (0.942, 0.997)	0.0300	0.995 (0.967, 1.023)	0.7122	0.995 (0.967, 1.024)	0.7365
SO_2_ (ppb)	0.991 (0.978, 1.005)	0.2181	1.009 (0.995, 1.022)	0.2155	1.009 (0.995, 1.023)	0.198
NO_2_ (ppb)	0.864 (0.828, 0.901)	< 0.0001	0.937 (0.886, 0.991)	0.0237	0.935 (0.884, 0.989)	0.0184
CO (ppb)	0.942 (0.907, 0.978)	0.002	1.037 (0.995, 1.081)	0.0819	1.036 (0.994, 1.08)	0.0964
**2-years**						
PM_10_ (µg/m^3^)	0.968 (0.936, 1.001)	0.0557	1.045 (1.009, 1.082)	0.0144	1.046 (1.009, 1.083)	0.0135
PM_2.5_ (µg/m^3^)	0.961 (0.933, 0.991)	0.0102	0.991 (0.961, 1.022)	0.5661	0.992 (0.962, 1.023)	0.6105
SO_2_ (ppb)	0.991 (0.977¸ 1.005)	0.2084	1.009 (0.995, 1.023)	0.1899	1.01 (0.996, 1.024)	0.1726
NO_2_ (ppb)	0.867 (0.831, 0.904)	< 0.0001	0.944 (0.893, 0.999)	0.0463	0.942 (0.889, 0.996)	0.0372
CO (ppb)	0.947 (0.912, 0.984)	0.0051	1.048 (1.006, 1.092)	0.0264	1.046 (1.004, 1.091)	0.0323

PM_10_, particulate matter ≤ 10 μm in diameter; PM_2.5,_ particulate matter ≤ 2.5 μm in diameter; SO_2_, sulfur dioxide; NO_2_, nitrogen dioxide; CO, carbon monoxide; CI, confidence interval

†Adjusted model 1 includes the following variables: place of residence (urban vs. rural), age, sex, household income, education level, alcohol consumption, smoking status, physical activity, and occupation

††Adjusted model 2 includes the following variables: place of residence (urban vs. rural), age, sex, household income, education level, alcohol consumption, smoking status, physical activity, occupation, hypertension status, diabetes status and obesity status (normal, overweight and obesity)

### Propensity score matching analysis results

We performed PSM analyses to address group imbalances between anemia and normal using 1:1 match ratio. A total of 12,496 individuals (6,248 per group) were included (Table S3). Compared to before PSM, the distributions of most characteristics after PSM were similar between anemia and the normal population, except for age, BMI, and hemoglobin level (all *p* > 0.05). The associations between ambient air pollution and hemoglobin level after PSM were shown in Table S4. In annual exposure model, exposure to most air pollutants, except for PM_2.5_, were significantly associated with a lower hemoglobin concentration (all *p* < 0.05). These results were similar for the association results during 2 years of exposure. However, in the association analysis with anemia, no significant association was observed for any air pollutants (all *p* > 0.05) (Table S5).

## Discussion

This study aimed to determine the associations between long-term exposure to air pollution and hemoglobin or anemia in general adults in Korea. We found that sustained exposure to ambient air pollution, especially PM_10_, SO_2_, and CO, was significantly associated with a decrease in hemoglobin concentrations. A significant association between NO_2_ exposure and decreased hemoglobin levels was observed only in men. Furthermore, long-term exposure to PM_10_ and CO was significantly associated with an increased risk of anemia. These findings highlight the importance of minimizing exposure to ambient air pollution in managing reduced hemoglobin levels or anemia in general adults.

Our study considered the various confounding factors such as demographic information, health-related behaviors, socio-economic level, and medical conditions. In fact, previous literature has reported that these confounding factors are closely associated with anemia. The prevalence of anemia gradually increases with age, especially among older adults over 60 years of age [24]. One recent study based on the multiethnic Iranian population showed that it was notably higher in women (17.08%) than men (4.87%), similar to our results [25]. Socioeconomic variables such as low family income and low maternal education level were also found to affect anemia in children [26, 27]. Besides, cigarette smoking affects the incidence of anemia as well as haematopoiesis [28] and poor physical activity was linked to increased risk of anemia [29]. In addition, participants’ health conditions, such as diabetes [29] and high blood pressure [30], were closely related to anemia. Similarly, in our study, significant statistical differences were found between anemia and normal groups in all the above potential variables. Therefore, we considered all these variables as confounding factors in the final analysis.

Previous epidemiological studies have identified significant relationships between exposure to ambient air pollutants and decreased hemoglobin or anemia, mainly in certain populations such as the elderly, children, and pregnant women [9–13, 31]. In 2017, Honda et al. reported that long-term exposure to PM_2.5_ and NO_2_ was positively associated with anemia prevalence and decreased hemoglobin levels in older American adults [10]. The prevalence ratio (95% CI) of anemia for each increase in IQR in PM_2.5_ and NO_2_ was 1.33 (1.23–1.45) and 1.43 (1.25–1.63), respectively. Similarly, a study based on a cohort of the Chinese elderly population showed that exposure to air pollutants, including PM_1_, PM_2.5_, PM_10_, and NO_2_, was closely related to an increased prevalence of anemia and decreased hemoglobin levels in a single pollutant model [9]. Furthermore, Poursafa et al. in 2011 found a significant negative association between PM_10_ and hemoglobin levels in children and adolescents [31]. Another study conducted among children aged 6–59 months living in Lima, Peru, found a significant association between outdoor PM_2.5_ levels and decreased hemoglobin concentrations or an increased prevalence of moderate/severe anemia [12]. The same results were found in Indian children under five years of age [11]. In addition, a recent study on pregnant women investigated the short-term effects of PM_2.5_ and its constituents (e.g., BC, NH_4_^+^, NO_3_^–^, OM, SO_4_^2–^, and dust) on anemia and hemoglobin levels during the third trimester. The study found that PM_2.5_ and some of its constituents were associated with a decrease in hemoglobin concentration rather than anemia [13]. Therefore, previous studies on specific vulnerable populations have shown significant associations between PM_2.5_ and NO_2_ and hemoglobin levels or anemia. These results are consistent with our findings in that several air pollutants are linked to a decreased hemoglobin level and an increased risk of anemia. However, no significance was observed for PM_2.5_ in our results. In the case of PM, especially PM_2.5_ has a smaller diameter than PM_10_, it can penetrate deeper into the lungs and the bloodstream [32]. For this reason, PM_2.5_ has been shown to be more closely related to negative health conditions than PM_10_. On the other hand, in our study, the significant association with PM_10_ rather than PM_2.5_ may suggest another important implication regardless of the size of the pollutant’s diameter. However, further research is needed to justify this. In addition, the discrepancies in results may be partly explained by differences in study design, study sample characteristics, ethnicity, exposure assessment/levels to air pollutants, geographic conditions, or pollutant composition.”

In addition, many studies have reported the associations of air pollution with peripheral blood cells and leukemia, which are closely related to hematopoietic function [33–41]. In 1999, Seaton et al. reported that PM_10_ exposure is associated with not only hemoglobin levels but also packed cell volume, and red blood cell (RBC) count among subjects aged over 60 years [38]. The negative effects of air pollution on red blood cells have also been found in children [36]. Likewise, a large-scale epidemiological study in eastern China observed that short-term exposure to PM_2.5_ was negatively associated with RBC count as well as hemoglobin levels [16, 41]. More recent studies showed that short- and long-term exposure to air pollution increases the risk of leukemia in adults as well as children [33–35, 37, 39, 40].

The mechanism underlying the relationship between ambient air pollution and changes in hemoglobin levels remains unclear, but several potential mechanisms have been suggested. Air pollutants directly or indirectly promote a chronic inflammatory process in the human body [42, 43]. Such systemic reactions can potentially contribute to changes in hemoglobin levels and an increased prevalence of anemia. A previous study on the association between air pollution and hematologic parameters found that the increase in PM_10_ quartiles was closely related to elevated white blood cell counts, as well as reduced hemoglobin concentration or RBC counts, highlighting the importance of the pro-inflammatory response [31]. Honda et al. (2017) also observed the mediating effect of C-reactive protein, a well-known indicator of systemic inflammation, in the association between NO_2_ or PM_2.5_ and hemoglobin [10]. Furthermore, inflammatory cytokines cause a deficiency in erythropoietin secretion and greater resistance to erythropoietin in the kidney [44, 45]. This induces a decrease in RBC counts and a lower hemoglobin concentration [44]. Furthermore, a high level of Interleukin 6, a pro-inflammatory cytokine, caused by exposure to air pollution can increase hepcidin production through the signal transducer and transcription activator-3, thus inducing iron deficiency [46, 47]. These hypotheses, based on chronic inflammation, support the impact of long-term exposure to air pollution on health outcomes such as anemia. However, additional studies are needed to prove the plausible hypotheses mentioned above.

For the first time, the present study reported that higher long-term exposures to PM_10_, SO_2_, and CO were significantly associated with reduced hemoglobin concentrations and an elevated risk of anemia in a nationally representative population of South Korea. Although previous studies have shown a significant association of air pollution with hemoglobin levels or anemia, most of them focused on certain vulnerable groups (e.g., the elderly, children, and pregnant women) or limited air pollutants such as PM and NO_2_. In this regard, the findings of our large-scale study on a general population may have important implications not only for specific vulnerable populations but also for general public health. Nationwide air pollution modeling data and survey data from South Korea used in our study can also minimize some biases that arise from regionally restricted studies. In addition, this study provides novel evidence for a significant relationship with other air pollutants, including SO_2_ and CO, in addition to previously known air pollutants, such as PM and NO_2_.

Despite the strengths of this study, several limitations need to be discussed. One of the major limitations is that information related to iron supplements or dietary intake could not be included in this study because of the absence of relevant data. Therefore, it is difficult to rule out the possible confounding effect of iron intake levels on our results. Given the lack of data, we also could not determine whether the women were pregnant at the time of the blood test. Furthermore, the design of this study was cross-sectional, and thus it is impossible to determine causality between exposure to air pollutants and the development of anemia. In addition, this study estimated individual exposure levels to outdoor air pollution by modeling based on the subjects’ current home addresses. Since there are no available data, we were unable to consider variables such as the level of exposure at work, exposure at a moving place, the indoor exposure, and the history of relocation. Lastly, the interpolation method used to increase the spatial resolution of PM_2.5_ and PM_10_ needs to be considered in a variety of way, such as random forest and deep learning, which is widely used.

In conclusion, our findings suggest that exposure to air pollution, even among adults in the general population of Korea, reduces hemoglobin levels and increases the risk of developing anemia. These results are the first clue for adults in general and may help shape future strategies to address air pollution exposure risks faced by the general population. However, more studies are needed to verify whether the relationship between air pollution exposure and changes in hemoglobin levels or anemia development is causal and whether nutritional interventions, such as iron intake, can be used to minimize the harmful effects of air pollution on anemia.

### Electronic supplementary material

Below is the link to the electronic supplementary material.

Supplementary Material 1

## Data Availability

The datasets used and/or analyzed during the current study available from the corresponding author on reasonable request.

## References

[1] Hunt JM (2002). Reversing productivity losses from iron deficiency: the economic case. J Nutr.

[2] Collaborators GBDA (2023). Prevalence, years lived with disability, and trends in anaemia burden by severity and cause, 1990–2021: findings from the global burden of Disease Study 2021. Lancet Haematol.

[3] Groenveld HF, Januzzi JL, Damman K, van Wijngaarden J, Hillege HL, van Veldhuisen DJ, van der Meer P (2008). Anemia and mortality in heart failure patients a systematic review and meta-analysis. J Am Coll Cardiol.

[4] Scicchitano P, Iacoviello M, Massari A, De Palo M, Potenza A, Landriscina R, Abruzzese S, Tangorra M, Guida P, Ciccone MM et al. Anaemia and Congestion in Heart Failure: Correlations and Prognostic Role. *Biomedicines* 2023, 11(3). 10.3390/biomedicines11030972 PMC10046168 36979951

[5] Stauder R, Valent P, Theurl I (2018). Anemia at older age: etiologies, clinical implications, and management. Blood.

[6] Endris BS, Dinant GJ, Gebreyesus SH, Spigt M (2022). Risk factors of anemia among preschool children in Ethiopia: a bayesian geo-statistical model. BMC Nutr.

[7] Gupta A, Ramakrishnan L, Pandey RM, Sati HC, Khandelwal R, Khenduja P, Kapil U (2020). Risk factors of anemia amongst elderly population living at high-altitude region of India. J Family Med Prim Care.

[8] Krishnapillai A, Omar MA, Ariaratnam S, Awaluddin S, Sooryanarayana R, Kiau HB, Tauhid NM, Ghazali SS. The prevalence of Anemia and its Associated factors among older persons: findings from the National Health and Morbidity Survey (NHMS) 2015. Int J Environ Res Public Health 2022, 19(9). 10.3390/ijerph19094983 PMC9101117 35564378

[9] Elbarbary M, Honda T, Morgan G, Guo Y, Guo Y, Kowal P, Negin J. Ambient Air Pollution Exposure Association with anaemia prevalence and haemoglobin levels in Chinese older adults. Int J Environ Res Public Health 2020, 17(9). 10.3390/ijerph17093209 PMC7246731 32380747

[10] Honda T, Pun VC, Manjourides J, Suh H (2017). Anemia prevalence and hemoglobin levels are associated with long-term exposure to air pollution in an older population. Environ Int.

[11] Mehta U, Dey S, Chowdhury S, Ghosh S, Hart JE, Kurpad A (2021). The Association between ambient PM(2.5) exposure and Anemia outcomes among children under five years of age in India. Environ Epidemiol.

[12] Morales-Ancajima VC, Tapia V, Vu BN, Liu Y, Alarcon-Yaquetto DE, Gonzales GF. Increased Outdoor PM(2.5) Concentration Is Associated with Moderate/Severe Anemia in Children Aged 6–59 Months in Lima, Peru. *J Environ Public Health* 2019, 2019:6127845. 10.1155/2019/6127845 PMC6681625 31428166

[13] Xie G, Yue J, Yang W, Yang L, Xu M, Sun L, Zhang B, Guo L, Chung MC (2022). Effects of PM(2.5) and its constituents on hemoglobin during the third trimester in pregnant women. Environ Sci Pollut Res Int.

[14] Ghio AJ, Soukup JM, Dailey LA, Madden MC (2020). Air pollutants disrupt iron homeostasis to impact oxidant generation, biological effects, and tissue injury. Free Radic Biol Med.

[15] Forbes LJ, Patel MD, Rudnicka AR, Cook DG, Bush T, Stedman JR, Whincup PH, Strachan DP, Anderson RH (2009). Chronic exposure to outdoor air pollution and markers of systemic inflammation. Epidemiology.

[16] Li W, Dorans KS, Wilker EH, Rice MB, Ljungman PL, Schwartz JD, Coull BA, Koutrakis P, Gold DR, Keaney JF (2017). Short-term exposure to Ambient Air Pollution and biomarkers of systemic inflammation: the Framingham Heart Study. Arterioscler Thromb Vasc Biol.

[17] Morceau F, Dicato M, Diederich M (2009). Pro-inflammatory cytokine-mediated anemia: regarding molecular mechanisms of erythropoiesis. Mediators Inflamm.

[18] van Eeden SF, Tan WC, Suwa T, Mukae H, Terashima T, Fujii T, Qui D, Vincent R, Hogg JC (2001). Cytokines involved in the systemic inflammatory response induced by exposure to particulate matter air pollutants (PM(10)). Am J Respir Crit Care Med.

[19] Nagababu E, Gulyani S, Earley CJ, Cutler RG, Mattson MP, Rifkind JM (2008). Iron-deficiency anaemia enhances red blood cell oxidative stress. Free Radic Res.

[20] Cho MH (2014). Are Korean adults meeting the recommendation for physical activity during Leisure Time?. J Phys Ther Sci.

[21] (WHO). WHO: Obesity and Overweight, Fact sheet, Updated. June 2016. http://wwww.hoint/mediacentre/factsheets/fs311/en. 2016.

[22] Lin Q, Ye T, Ye P, Borghi C, Cro S, Damasceno A, Khan N, Nilsson PM, Prabhakaran D, Ramirez A (2022). Hypertension in stroke survivors and associations with national premature stroke mortality: data for 2.5 million participants from multinational screening campaigns. Lancet Glob Health.

[23] Donnelly JP, Nair S, Griffin R, Baddley JW, Safford MM, Wang HE, Shapiro NI (2017). Association of Diabetes and insulin therapy with risk of hospitalization for infection and 28-Day mortality risk. Clin Infect Dis.

[24] Guralnik J, Ershler W, Artz A, Lazo-Langner A, Walston J, Pahor M, Ferrucci L, Evans WJ (2022). Unexplained anemia of aging: etiology, health consequences, and diagnostic criteria. J Am Geriatr Soc.

[25] Akbarpour E, Paridar Y, Mohammadi Z, Mard A, Danehchin L, Abolnezhadian F, Azadpour S, Rahimi Z, Zamani M, Cheraghian B (2022). Anemia prevalence, severity, types, and correlates among adult women and men in a multiethnic Iranian population: the Khuzestan Comprehensive Health Study (KCHS). BMC Public Health.

[26] Bayoumi I, Parkin PC, Birken CS, Maguire JL, Borkhoff CM, Collaboration TAK (2020). Association of Family Income and Risk of Food Insecurity with Iron Status in Young Children. JAMA Netw Open.

[27] Oliveira MA, Osorio MM, Raposo MC (2007). Socioeconomic and dietary risk factors for anemia in children aged 6 to 59 months. J Pediatr (Rio J).

[28] Leifert JA (2008). Anaemia and cigarette smoking. Int J Lab Hematol.

[29] Zamani M, Poustchi H, Shayanrad A, Pourfarzi F, Farjam M, Noemani K, Ghaderi E, Mohammadkarimi V, Kahnooji M, Mansour-Ghanaei F (2022). Prevalence and determinants of anemia among Iranian population aged >/=35 years: a PERSIAN cohort-based cross-sectional study. PLoS ONE.

[30] Gela YY, Belay DG, Chilot D, Andualem AA, Bitew DA, Sinamaw D, Eshetu HB, Seid AM, Simegn W, Kibret AA (2023). Prevalence of anemia and associated factors among adult hypertensive patients in Referral hospitals, Amhara Regional State. Sci Rep.

[31] Poursafa P, Kelishadi R, Amini A, Amini A, Amin MM, Lahijanzadeh M, Modaresi M (2011). Association of air pollution and hematologic parameters in children and adolescents. J Pediatr (Rio J).

[32] Lee BJ, Kim B, Lee K (2014). Air pollution exposure and cardiovascular disease. Toxicol Res.

[33] Filippini T, Hatch EE, Rothman KJ, Heck JE, Park AS, Crippa A, Orsini N, Vinceti M (2019). Association between Outdoor Air Pollution and Childhood Leukemia: a systematic review and dose-response Meta-analysis. Environ Health Perspect.

[34] Khorrami Z, Pourkhosravani M, Eslahi M, Rezapour M, Akbari ME, Amini H, Taghavi-Shahri SM, Kunzli N, Etemad K, Khanjani N (2022). Multiple air pollutants exposure and leukaemia incidence in Tehran, Iran from 2010 to 2016: a retrospective cohort study. BMJ Open.

[35] Kreis C, Heritier H, Scheinemann K, Hengartner H, de Hoogh K, Roosli M, Spycher BD (2022). Childhood cancer and traffic-related air pollution in Switzerland: a nationwide census-based cohort study. Environ Int.

[36] Nikolic M, Nikic D, Stankovic A (2008). Effects of Air Pollution on Red Blood cells in children. Pol J Environ.

[37] Puett RC, Poulsen AH, Taj T, Ketzel M, Geels C, Brandt J, Christensen JH, Sorensen M, Roswall N, Hvidtfeldt U (2020). Relationship of leukaemias with long-term ambient air pollution exposures in the adult Danish population. Br J Cancer.

[38] Seaton A, Soutar A, Crawford V, Elton R, McNerlan S, Cherrie J, Watt M, Agius R, Stout R (1999). Particulate air pollution and the blood. Thorax.

[39] Taj T, Poulsen AH, Ketzel M, Geels C, Brandt J, Christensen JH, Puett R, Hvidtfeldt UA, Sorensen M, Raaschou-Nielsen O (2021). Exposure to PM(2.5) constituents and risk of adult leukemia in Denmark: a population-based case-control study. Environ Res.

[40] Xue Y, Cong J, Bai Y, Zheng P, Hu G, Kang Y, Wu Y, Cui L, Jia G, Wang T. Associations between Short-Term Air Pollution exposure and the peripheral leukocyte distribution in the Adult Male Population in Beijing, China. Int J Environ Res Public Health 2023, 20(6). 10.3390/ijerph20064695 PMC10048523 36981603

[41] Li Z, Li X, Song H, Qiu B, Tian D, Zhan M, Wu Z, Wu J, Zhang Q, Wang J (2021). Effects of short-term ambient PM2.5 exposure on the blood cell count and hemoglobin concentration among 82,431 people in eastern China. Sci Total Environ.

[42] Tripathy S, Marsland AL, Kinnee EJ, Tunno BJ, Manuck SB, Gianaros PJ, Clougherty JE (2021). Long-term Ambient Air Pollution exposures and circulating and stimulated inflammatory mediators in a cohort of midlife adults. Environ Health Perspect.

[43] Zeka A, Sullivan JR, Vokonas PS, Sparrow D, Schwartz J (2006). Inflammatory markers and particulate air pollution: characterizing the pathway to disease. Int J Epidemiol.

[44] Barany P (2001). Inflammation, serum C-reactive protein, and erythropoietin resistance. Nephrol Dial Transpl.

[45] Gluba-Brzozka A, Franczyk B, Olszewski R, Rysz J. The influence of inflammation on Anemia in CKD patients. Int J Mol Sci 2020, 21(3). 10.3390/ijms21030725 PMC7036805 31979104

[46] Wrighting DM, Andrews NC. Interleukin-6 induces hepcidin expression through STAT3. *Blood* 2006, 108(9):3204–3209. 10.1182/blood-2006-06-027631 PMC1895528 16835372

[47] Yacoub MF, Ferwiz HF, Said F. Effect of Interleukin and Hepcidin in Anemia of Chronic Diseases. *Anemia* 2020, 2020:3041738. 10.1155/2020/3041738 PMC7033950 32095285

