# Supplementary material for: Association of ambient air pollution with hemoglobin levels and anemia in the general population of Korean adults
Source: BMC Public Health. 2024 Apr 9;24:988. doi: 10.1186/s12889-024-18492-z (PMC11003135; doi:10.1186/s12889-024-18492-z)
Supplement: Supplementary file 1 — Supplementary Material 1 [file 12889_2024_18492_MOESM1_ESM.docx]

**Supplementary Files**


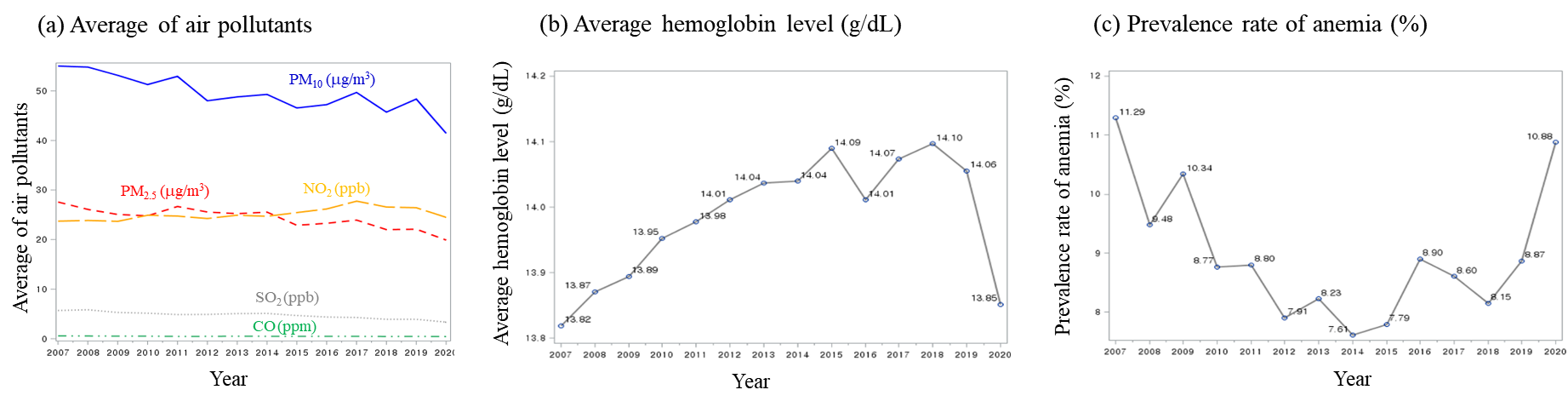


Figure S1. Average levels of each air pollutant and hemoglobin level, and prevalence of anemia by year (2007-2020)


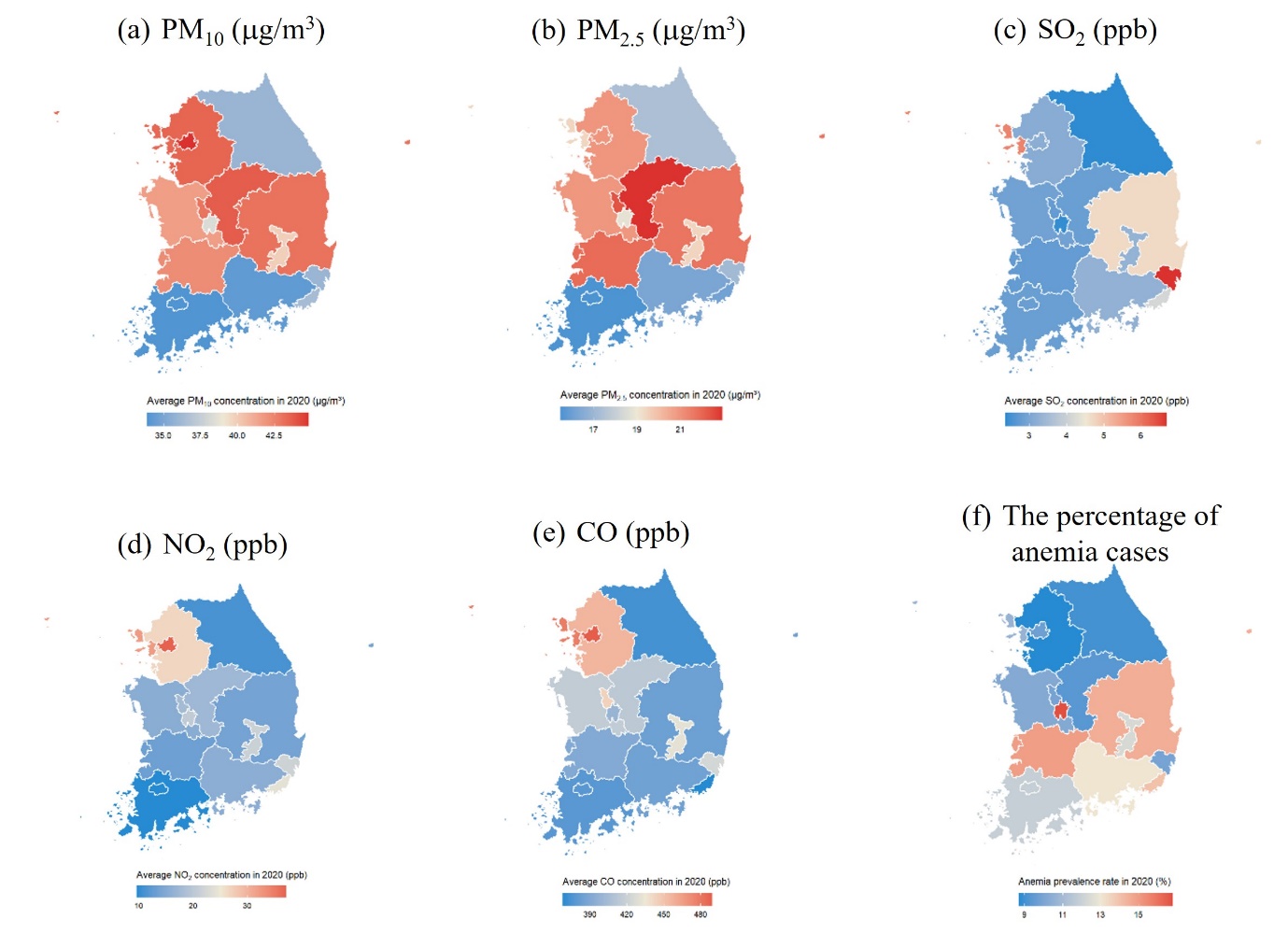


Figure S2. Distribution of air pollution concentration (a) ~ (e) and anemia incidence rate (f) by region in Korea for the most recent year (2020)

Table S1. Association between air pollution and hemoglobin levels, stratified by sex

|  | **Log (Hemoglobin level)** | | | | | | | |
| --- | --- | --- | --- | --- | --- | --- | --- | --- |
|  | **Crude model** | |  | **Adjusted model 1**^†^ | |  | **Adjusted model 2**^† †^ | |
|  | *β* (95% CI) | *p* |  | *β* (95% CI) | *p* |  | *β* (95% CI) | *p* |
| **1-year** |  |  |  |  |  |  |  |  |
| **Women (n= 39,503)** | |  |  |  |  |  |  |  |
| PM_10_ (μg/m^3^) | -0.0032 (-0.0053, -0.0011) | 0.0027 |  | -0.0033 (-0.0055, -0.0012) | 0.0024 |  | -0.0032 (-0.0053, -0.001) | 0.0037 |
| PM_2.5_ (μg/m^3^) | 0.0007 (-0.0009, 0.0024) | 0.3915 |  | 0.0005 (-0.0012, 0.0022) | 0.543 |  | 0.0006 (-0.001, 0.0023) | 0.469 |
| SO_2_ (ppb) | -0.0012 (-0.002, -0.0004) | 0.0026 |  | -0.0015 (-0.0022, -0.0006) | 0.0004 |  | -0.0014 (-0.0022, -0.0006) | 0.0006 |
| NO_2_ (ppb) | 0.0012 (-0.0013, 0.0036) | 0.3574 |  | 0.0033 (0.0001, 0.0065) | 0.042 |  | 0.0036 (0.0004, 0.0068) | 0.0273 |
| CO (ppb) | -0.0044 (-0.0066, -0.0022) | 0.0001 |  | -0.0046 (-0.007, -0.0023) | 0.0001 |  | -0.0044 (-0.0068, -0.002) | 0.0003 |
| **Men (n= 30,327)** | |  |  |  |  |  |  |  |
| PM_10_ (μg/m^3^) | 0.0046 (0.0021, 0.007) | 0.0003 |  | -0.004 (-0.0064, -0.0016) | 0.0013 |  | -0.0035 (-0.0059, -0.0012) | 0.0036 |
| PM_2.5_ (μg/m^3^) | 0.005 (0.003, 0.0069) | <0.0001 |  | 0.0019 (0, 0.0037) | 0.0417 |  | 0.0023 (0.00046, 0.0041) | 0.0141 |
| SO_2_ (ppb) | 0.0009 (0, 0.0019) | 0.0526 |  | -0.0012 (-0.0021, -0.0003) | 0.0083 |  | -0.0011 (-0.002, -0.0002) | 0.0129 |
| NO_2_ (ppb) | 0.0104 (0.0075, 0.0133) | <0.0001 |  | -0.0061 (-0.0096, -0.0026) | 0.0006 |  | -0.0062 (-0.0097, -0.0027) | 0.0004 |
| CO (ppb) | 0.0074 (0.0048, 0.01) | <0.0001 |  | -0.0055 (-0.0082, -0.0028) | <0.0001 |  | -0.0052 (-0.0078, -0.0026) | 0.0001 |
| **2-year** |  |  |  |  |  |  |  |  |
| **Women** | |  |  |  |  |  |  |  |
| PM_10_ (μg/m^3^) | -0.0044 (-0.0063, -0.0024) | <0.0001 |  | -0.0046 (-0.0066, -0.0025) | <0.0001 |  | -0.0045 (-0.0065, -0.0024) | <0.0001 |
| PM_2.5_ (μg/m^3^) | 0.0006 (-0.0012, 0.0024) | 0.5166 |  | 0.0003 (-0.0015, 0.0021) | 0.7197 |  | 0.0004 (-0.0014, 0.0022) | 0.6468 |
| SO_2_ (ppb) | -0.0013 (-0.0021, -0.0005) | 0.0021 |  | -0.0015 (-0.0023, -0.0007) | 0.0004 |  | -0.0015 (-0.0023, -0.0006) | 0.0004 |
| NO_2_ (ppb) | 0.0008 (-0.0017, 0.0033) | 0.5294 |  | 0.0029 (-0.0004, 0.0061) | 0.0844 |  | 0.0031 (-0.0001, 0.0063) | 0.0582 |
| CO (ppb) | -0.0053 (-0.0075, -0.0031) | <0.0001 |  | -0.0056 (-0.008., -0.0032) | <0.0001 |  | -0.0054 (-0.0078, -0.003) | <0.0001 |
| **Men** | |  |  |  |  |  |  |  |
| PM_10_ (μg/m^3^) | 0.0041 (0.0018, 0.0064) | 0.0004 |  | -0.0043 (-0.0066, -0.002) | 0.0002 |  | -0.004 (-0.0063, -0.0017) | 0.0005 |
| PM_2.5_ (μg/m^3^) | 0.005 (0.0032, 0.0073) | <0.0001 |  | 0.0019 (0, 0.0039) | 0.0559 |  | 0.0022 (0.0003, 0.0042) | 0.0244 |
| SO_2_ (ppb) | 0.0009 (0, 0.0019) | 0.0611 |  | -0.0013 (-0.0022, -0.0004) | 0.0058 |  | -0.0012 (-0.0021, -0.0003) | 0.0079 |
| NO_2_ (ppb) | 0.0103 (0.0074, 0.0132) | <0.0001 |  | -0.0062 (-0.0097, -0.0027) | 0.0005 |  | -0.0064 (-0.0099, -0.0029) | 0.0003 |
| CO (ppb) | 0.0073 (0.0047, 0.0099) | <0.0001 |  | -0.0059 (-0.0085, -0.0032) | <0.0001 |  | -0.0056 (-0.0082, -0.0029) | <0.0001 |

PM_10_, particulate matter ≤10 μm in diameter; PM_2.5,_ particulate matter ≤ 2.5 μm in diameter; SO_2_, sulfur dioxide; NO_2_, nitrogen dioxide; CO, carbon monoxide; CI, confidence interval.

†Adjusted model 1 includes the following variables: place of residence (urban vs. rural), age, sex, household income, education level, alcohol consumption, smoking status, physical activity and occupation.

††Adjusted model 2 includes the following variables: place of residence (urban vs. rural), age, sex, household income, education level, alcohol consumption, smoking status, physical activity, occupation, hypertension status, diabetes status and obesity status (normal, overweight and obesity).

Table S2. Association between air pollution and anemia status, stratified by sex

|  | **Anemia status** | | | | | | | |
| --- | --- | --- | --- | --- | --- | --- | --- | --- |
|  | **Crude model** | |  | **Adjusted model 1**^†^ | |  | **Adjusted model 2**^† †^ | |
|  | *OR* (95% CI) | *p* |  | *OR* (95% CI) | *p* |  | *OR* (95% CI) | *p* |
| **1-year** |  |  |  |  |  |  |  |  |
| **Women (n= 39,503)** | |  |  |  |  |  |  |  |
| PM_10_ (μg/m^3^) | 1.011 (0.971, 1.053) | 0.5883 |  | 1.037 (0.994, 1.081) | 0.0922 |  | 1.037 (0.994, 1.081) | 0.0951 |
| PM_2.5_ (μg/m^3^) | 0.997 (0.965, 1.029) | 0.8417 |  | 1.002 (0.970, 1.035) | 0.913 |  | 1.002 (0.97, 1.035) | 0.8927 |
| SO_2_ (ppb) | 0.999 (0.984, 1.015) | 0.9207 |  | 1.007 (0.991, 1.022) | 0.3918 |  | 1.007 (0.992, 1.023) | 0.3775 |
| NO_2_ (ppb) | 0.912 (0.869, 0.957) | 0.0002 |  | 0.929 (0.872, 0.99) | 0.0235 |  | 0.926 (0.869, 0.986) | 0.0172 |
| CO (ppb) | 1.010 (0.967, 1.055) | 0.64 |  | 1.046 (0.999, 1.096) | 0.0562 |  | 1.045 (0.997, 1.095) | 0.0661 |
| **Men (n= 30,327)** | |  |  |  |  |  |  |  |
| PM_10_ (μg/m^3^) | 0.802 (0.742, 0.866) | <0.0001 |  | 0.989 (0.912, 1.071) | 0.7806 |  | 0.988 (0.911, 1.071) | 0.7694 |
| PM_2.5_ (μg/m^3^) | 0.873 (0.822, 0.928) | <0.0001 |  | 0.936 (0.881, 0.995) | 0.995 |  | 0.934 (0.878, 0.993) | 0.0284 |
| SO_2_ (ppb) | 0.944 (0.913, 0.976) | 0.0006 |  | 1.007 (0.978, 1.037) | 0.6327 |  | 1.008 (0.979, 1.037) | 0.6146 |
| NO_2_ (ppb) | 0.681 (0.622, 0.747) | <0.0001 |  | 1.011 (0.895, 1.141) | 0.8639 |  | 1.01 (0.894, 1.142) | 0.8692 |
| CO (ppb) | 0.722 (0.665, 0.783) | <0.0001 |  | 0.964 (0.88, 1.056) | 0.4271 |  | 0.955 (0.871, 1.047) | 0.3269 |
| **2-year** |  |  |  |  |  |  |  |  |
| **Women** | |  |  |  |  |  |  |  |
| PM_10_ (μg/m^3^) | 1.018 (0.980. 1.058) | 0.3563 |  | 1.044 (1.003, 1.086) | 0.0348 |  | 1.044(1.003, 1.086) | 0.035 |
| PM_2.5_ (μg/m^3^) | 0.991 (0.957, 1.025) | 0.5952 |  | 0.997 (0.963, 1.032) | 0.8551 |  | 0.998 (0.963, 1.033) | 0.8905 |
| SO_2_ (ppb) | 0.999 (0.984, 1.015) | 0.9247 |  | 1.007 (0.992, 1.023) | 0.365 |  | 1.008 (0.992, 1.024) | 0.3504 |
| NO_2_ (ppb) | 0.917 (0.873, 0.963) | 0.0005 |  | 0.937 (0.879, 0.999) | 0.0478 |  | 0.934 (0.876, 0.996) | 0.0359 |
| CO (ppb) | 1.021 (0.978, 1.067) | 0.3396 |  | 1.059 (1.011, 1.109) | 0.0158 |  | 1.057 (1.009, 1.108) | 0.0194 |
| **Men** | |  |  |  |  |  |  |  |
| PM_10_ (μg/m^3^) | 0.794 (0.739, 0.854) | <0.0001 |  | 0.979 (0.907, 1.057) | 0.5888 |  | 0.979 (0.906, 1.058) | 0.6002 |
| PM_2.5_ (μg/m^3^) | 0.859 (0.805, 0.917) | <0.0001 |  | 0.927 (0.868, 0.989) | 0.0214 |  | 0.925 (0.866, 0.987) | 0.0191 |
| SO_2_ (ppb) | 0.942 (0.911, 0.975) | 0.0006 |  | 1.008 (0.979, 1.038) | 0.5941 |  | 1.009 (0.979, 1.039) | 0.5628 |
| NO_2_ (ppb) | 0.679 (0.619, 0.744) | <0.0001 |  | 1.009 (0.893, 1.14) | 0.8907 |  | 1.009 (0.892, 1.142) | 0.8826 |
| CO (ppb) | 0.715 (0.659, 0.776) | <0.0001 |  | 0.963 (0.879, 1.055) | 0.4208 |  | 0.955 (0.871, 1.048) | 0.3332 |

PM_10_, particulate matter ≤10 μm in diameter; PM_2.5,_ particulate matter ≤ 2.5 μm in diameter; SO_2_, sulfur dioxide; NO_2_, nitrogen dioxide; CO, carbon monoxide; CI, confidence interval.

†Adjusted model 1 includes the following variables: place of residence (urban vs. rural), age, sex, household income, education level, alcohol consumption, smoking status, physical activity and occupational group.

††Adjusted model 2 includes the following variables: place of residence (urban vs. rural), age, sex, household income, education level, alcohol consumption, smoking status, physical activity, occupational group, hypertension status, diabetes status and obesity status (normal, overweight and obesity).

Table S3. Baseline characteristics by anemia before and after propensity score (PS) matching

| **Characteristics** | **Before PS match** | | |  | **After PS match** | | |
| --- | --- | --- | --- | --- | --- | --- | --- |
|  | **Anemia**  (Women: Hemoglobin level < 12g/dL)  (Men: Hemoglobin level < 13g/dL) | **Normal**  (Women: Hemoglobin level ≥ 12g/dL)  (Men: Hemoglobin level ≥ 13g/dL) | *p* |  | **Anemia**  (Women: Hemoglobin level < 12g/dL)  (Men: Hemoglobin level < 13g/dL) | **Normal**  (Women: Hemoglobin level ≥ 12g/dL)  (Men: Hemoglobin level ≥ 13g/dL) | *p* |
|  | Mean ± SD or n (%) | Mean ± SD or n (%) |  |  | Mean ± SD or n (%) | Mean ± SD or n (%) |  |
| **n** | 6,248 (9.0) | 63,582 (91.1) |  |  | 6,248 (50.0) | 6,248 (50.0) |  |
| **Age**(year) | 54.2 ± 17.2 | 50.1 ± 16.0 | <.0001 |  | 54.2± 17.2 | 52.5± 16.6 | <.0001 |
| **Sex** |  |  | <.0001 |  |  |  | 0.7412 |
| Women | 4,937 (79.0) | 34,566 (54.4) |  |  | 4,937 (79.0.) | 4,952 (79.3) |  |
| Men | 1,311 (21.0) | 29,016 (45.6) |  |  | 1,311 (21.0) | 1,296 (20.7) |  |
| **Education level** |  |  | <.0001 |  |  |  | 0.9974 |
| Less than elementary school | 1,929 (30.9) | 13,951 (21.9) |  |  | 1929 (30.9) | 1,935 (31) |  |
| Middle school | 624 (10.0) | 6,777 (10.7) |  |  | 624 (10.0) | 625 (10) |  |
| High school | 1,911 (30.6) | 21,347 (33.6) |  |  | 1911 (30.6) | 1900 (30.4) |  |
| College or graduate school | 1,784 (28.6) | 21,507 (33.8) |  |  | 1784 (28.6) | 1788 (28.6) |  |
| [**Residential**](https://en.dict.naver.com/#/entry/enko/ef70c27bd626408ca9847fa2482822d9)**region** |  |  | <.0001 |  |  |  | 0.7135 |
| Rural | 1,952 (31.2) | 17,628 (27.7) |  |  | 1,952 (31.2) | 1,933 (30.9) |  |
| Urban | 4,296 (68.8) | 45,954 (72.3) |  |  | 4,296 (68.8) | 4,315 (69.1) |  |
| **Smoking** |  |  | <.0001 |  |  |  | 0.9772 |
| Never or Former-smokers | 5,558 (89.0) | 47,553 (74.8) |  |  | 5,558 (89.0) | 5,557 (88.9) |  |
| Current smokers | 690 (11.0) | 16,029 (25.2) |  |  | 690 (11.0) | 691 (11.1) |  |
| **Alcohol consumption**  (time/month) |  |  | <.0001 |  |  |  | 0.9829 |
| Never | 2,497 (40.0) | 16,869 (26.5) |  |  | 2,497 (40.0) | 2,494 (39.9) |  |
| ≤ 1 | 1,949 (31.2) | 18,102 (28.5) |  |  | 1,949 (31.2) | 1,946 (31.2) |  |
| 2~15 | 1,572 (25.2) | 24,056 (37.8) |  |  | 1,572 (25.2) | 1,585 (25.4) |  |
| ≥ 16 | 230 (3.7) | 4,555 (7.2) |  |  | 230 (3.7) | 223 (3.6) |  |
| **Physical activity** |  |  | <.0001 |  |  |  | 0.6449 |
| Yes | 1,325 (21.2) | 15,704 (24.7) |  |  | 1325 (21.2) | 1304 (20.9) |  |
| No | 4,923 (78.8) | 47,878 (75.3) |  |  | 4923 (78.8) | 4944 (79.1) |  |
| **Household income** (Quartile, Thousand KRW) |  |  | <.0001 |  |  |  | 0.9885 |
| Low (< 76) | 1,623 (26.0) | 11,392 (17.9) |  |  | 1,623 (26.0) | 1,613 (25.8) |  |
| Lower-medium (76~154) | 1,583 (25.3) | 15,837 (24.9) |  |  | 1,583 (25.3) | 1,598 (25.6) |  |
| Upper-medium (155~253) | 1,598 (25.6) | 17,536 (27.6) |  |  | 1,598 (25.6) | 1,601 (25.6) |  |
| High (≥ 254) | 1,444 (23.1) | 18,817 (29.6) |  |  | 1,444 (23.1) | 1,436 (23.0) |  |
| **Occupational group** |  |  | <.0001 |  |  |  | 0.9747 |
| Office worker | 614 (9.8) | 8,207 (12.9) |  |  | 614 (33.6) | 603 (33.2) |  |
| Service/sales worker | 416 (6.7) | 4,644 (7.3) |  |  | 416 (22.8) | 413 (22.8) |  |
| Skilled agricultural, forestry, fishery workers | 282 (4.5) | 2,684 (4.2) |  |  | 282 (15.4) | 290 (16.0) |  |
| Craft and related trades workers/plant, machine operators and assemblers/ elementary workers | 514 (8.2) | 7,030 (11.1) |  |  | 514 (28.2) | 508 (28.00) |  |
| Unemployed/ unknown | 4,422 (70.8) | 41,017 (64.5) |  |  | - | - |  |
| **Body mass index** (kg/m^2^) | 22.9 ± 3.3 | 23.9 ± 3.4 | <.0001 |  | 23.2± 3.2 | 22.9 ± 3.3 | <.0001 |
| **Obesity status** |  |  | <.0001 |  |  |  | 0.8568 |
| Normal | 4,771 (76.4) | 41,828 (65.8) |  |  | 4,771 (76.4) | 4,772 (76.4) |  |
| Overweight | 1,292 (20.7) | 18,739 (29.5) |  |  | 1,292 (20.7) | 1,301 (20.8) |  |
| Obesity | 185 (3.0) | 3,015 (4.7) |  |  | 185 (3.0) | 175 (2.80) |  |
| **Hypertension status** |  |  | <.0001 |  |  |  | 0.7765 |
| Yes | 2,114 (33.8) | 19,696 (31.0) |  |  | 2,114 (33.8) | 2,099 (33.6) |  |
| No | 4,134 (66.2) | 43,886 (69.0) |  |  | 4,134 (66.2) | 4,149 (66.4) |  |
| **Diabetes status** |  |  | <.0001 |  |  |  | 0.5575 |
| Yes | 1,008 (16.1) | 7,106 (11.2) |  |  | 1,008 (16.1) | 984 (15.8) |  |
| No | 5,240 (83.9) | 56,476 (88.8) |  |  | 5,240 (83.9) | 5,264 (84.3) |  |
| **Hemoglobin level (g/dL)** | 11.3 ± 0.9 | 14.3 ± 1.3 | <.0001 |  | 11.3 ± 1.0 | 13.7 ± 1.0 | <.0001 |

SD, standard deviation, PS, propensity score

Table S4. Association between hemoglobin levels (g/dL) and interquartile range (IQR) of annual average air pollution exposure using propensity score (PS) matching

| **Exposure** | **Hemoglobin level** | | | | |
| --- | --- | --- | --- | --- | --- |
|  | **Before PS match (*n*= 69,830)** ^†^ | |  | **After PS match (*n*= 12,496)** | |
|  | *β* (95% CI) | *p* |  | *β* (95% CI) | *p* |
| **1-year** |  |  |  |  |  |
| PM_10_ (μg/m^3^) | -0.0043 (-0.0059, -0.0027) | < 0.0001 |  | -0.009 (-0.0143, -0.0038) | 0.0008 |
| PM_2.5_ (μg/m^3^) | 0.0006 (-0.0007, 0.0018) | 0.3566 |  | -0.0002 (-0.0044. 0.004) | 0.9137 |
| SO_2_ (ppb) | -0.0015 (-0.002, -0.0009) | < 0.0001 |  | -0.0028 (-0.0049, -0.0008) | 0.0070 |
| NO_2_ (ppb) | 0 (-0.0023, 0.0025) | 0.9432 |  | -0.0102 (-0.0164, -0.0039) | 0.0015 |
| CO (ppb) | -0.0054 (-0.0072, -0.0036) | <0.0001 |  | -0.0119 (-0.0176, -0.0063) | <0.0001 |
|  |  |  |  |  |  |
| **2-years** |  |  |  |  |  |
| PM_10_ (μg/m^3^) | -0.0054 (-0.0069, -0.0038) | < 0.0001 |  | -0.0107 (-0.0156, -0.0057) | <.0001 |
| PM_2.5_ (μg/m^3^) | 0.0002 (-0.0011, 0.0016) | 0.7361 |  | -0.0005 (-0.005, 0.0039) | 0.82 |
| SO_2_ (ppb) | -0.0016 (-0.0023, -0.001) | < 0.0001 |  | -0.0029 (-0.005, -0.0008) | 0.0075 |
| NO_2_ (ppb) | -0.0003 (-0.0027, 0.0021) | 0.8019 |  | -0.0112 (-0.0175, -0.0049) | 0.0005 |
| CO (ppb) | -0.0062 (-0.008, -0.0044) | <0.0001 |  | -0.0134 (-0.0191, -0.0078) | <0.0001 |

PM_10,_ particulate matter <10 μm in diameter; PM_2.5,_ particulate matter < 2.5 μm in diameter; SO_2_, sulfur dioxide; NO_2_, nitrogen dioxide; CO, carbon monoxide; CI: confidence interval.

†Before Propensity Score (PS) Matching model, an adjusted model that includes the following variables: place of residence (urban vs. rural), age, sex, household income, education level, alcohol consumption, smoking status, physical activity, occupational group, hypertension status, diabetes status, and obesity status (normal, overweight, and obesity).

Table S5. Association between anemia status and interquartile range (IQR) in annual average air pollution exposure using propensity score (PS) matching

| **Exposure** | **Anemia status** | | | | |
| --- | --- | --- | --- | --- | --- |
|  | **Before PS match (*n*= 69,830)** ^†^ | |  | **After PS match (*n*= 12,496)** | |
|  | *OR* (95% *CI*) | *p* |  | *OR* (95% *CI*) | *p* |
| **1-year** |  |  |  |  |  |
| PM_10_ (μg/m^3^) | 1.039 (1.001, 1.079) | 0.0426 |  | 1.026 (0.979, 1.076) | 0.2854 |
| PM_2.5_ (μg/m^3^) | 0.995 (0.967, 1.024) | 0.7365 |  | 0.987 (0.951. 1.025) | 0.5036 |
| SO_2_ (ppb) | 1.009 (0.995, 1.023) | 0.198 |  | 1.013 (0.994, 1.032) | 0.1833 |
| NO_2_ (ppb) | 0.935 (0.884, 0.989) | 0.0184 |  | 0.976 (0.923, 1.032) | 0.3981 |
| CO (ppb) | 1.036 (0.994, 1.08) | 0.0964 |  | 1.028 (0.977, 1.081) | 0.2899 |
|  |  |  |  |  |  |
| **2-years** |  |  |  |  |  |
| PM_10_ (μg/m^3^) | 1.046 (1.009, 1.083) | 0.0135 |  | 1.044 (0.999, 1.092) | 0.0544 |
| PM_2.5_ (μg/m^3^) | 0.992 (0.962, 1.023) | 0.6105 |  | 0.991 (0.952, 1.031) | 0.6420 |
| SO_2_ (ppb) | 1.01 (0.996, 1.024) | 0.1726 |  | 1.013 (0.994, 1.032) | 0.1779 |
| NO_2_ (ppb) | 0.942 (0.889, 0.996) | 0.0372 |  | 0.984 (0.93, 1.041) | 0.5811 |
| CO (ppb) | 1.046 (1.004, 1.091) | 0.0323 |  | 1.041 (0.989, 1.095) | 0.1232 |

PM_10,_ particulate matter <10 μm in diameter; PM_2.5,_ particulate matter < 2.5 μm in diameter; SO_2_, sulfur dioxide; NO_2_, nitrogen dioxide; CO, carbon monoxide; CI: confidence interval.

†Before Propensity Score (PS) Matching model, an adjusted model that includes the following variables: place of residence (urban vs. rural), age, sex, household income, education level, alcohol consumption, smoking status, physical activity, occupational group, hypertension status, diabetes status, and obesity status (normal, overweight, and obesity).
